# Endocrine correlates of male breast cancer risk: a case-control study in Athens, Greece

**DOI:** 10.1054/bjoc.2000.1467

**Published:** 2000-11

**Authors:** E Petridou, G Giokas, H Kuper, L A Mucci, D Trichopoulos

**Affiliations:** Department of Epidemiology, Harvard School of Public Health, Boston, MA, USA; Department of Hygiene and Epidemiology, University of Athens Medical School, Goudi, Athens, Greece

**Keywords:** male breast cancer, smoking, birth order, endocrine factors

## Abstract

We studied the relation of certain endocrine-related variables among 23 cases of male breast cancer and 76 apparently healthy male controls. There were significant inverse associations with smoking (*P* = 0.03), birth order (*P* = 0.02) and reported frequency of orgasms in later life (*P* = 0.0004). The study provides strong indirect evidence that endocrine factors are important in the aetiology of male breast cancer. © 2000 Cancer Research Campaign


*British Journal of Cancer* (2000) **83**(9), 1234–1237
© 2000 Cancer Research Campaign
doi: 10.1054/ bjoc.2000.1467, available online at http://www.idealibrary.com on IDEAL®

Short Communication

### Endocrine correlates of male breast cancer risk: a case-control study in Athens, Greece

**E Petridou[1,2], G Giokas[2], H Kuper[1], LA Mucci[1] and D Trichopoulos[1,2]**

[1]Department of Epidemiology, Harvard School of Public Health, Boston, MA, USA; [2]Department of Hygiene and Epidemiology, University of Athens Medical School, Goudi, Athens, Greece

**Summary** We studied the relation of certain endocrine-related variables among 23 cases of male breast cancer and 76 apparently healthy male controls. There were significant inverse associations with smoking ($P$ = 0.03), birth order ($P$ = 0.02) and reported frequency of orgasms in later life ($P$ = 0.0004). The study provides strong indirect evidence that endocrine factors are important in the aetiology of male breast cancer. © 2000 Cancer Research Campaign

**Keywords**: amale breast cancer; smoking; birth order; endocrine factors

Understanding the aetiology of male breast cancer could contribute towards an understanding of the disease among women. The principal findings in previous studies were that male breast cancer is more common among unmarried and childless men as well as among those with gynecomastia.

The collective epidemiological evidence suggests that increased oestrogen production and/or sexual drive-reducing endocrine conditions could be the underlying hormonal pattern in male breast cancer (Rose, 1988; Thomas et al, 1992). This is supported by a retrospective cohort study in Denmark indicating an excess occurrence of breast cancer among men with cirrhosis, who are considered as having elevated values of blood oestrogens (Sorensen et al, 1998), in keeping with the role of oestrogens in female breast cancer (Toniolo et al, 1995; Collaborative Group on Hormonal Factors in Breast Cancer, 1996, 1997; Adami et al, 1998; Hankinson et al, 1998).

We have undertaken a case-control study of male breast cancer in Athens, Greece, with the following objectives: (a) to evaluate the role of sexual drive, as indicated by the frequency of orgasms, whether through intercourse or other means (Mantzoros et al, 1995; McClellan and Markham, 1999); (b) to assess whether tobacco smoking may be inversely related to risk since tobacco smoking adversely affects oestrogenicity (Michnovicz and Fishman, 1990); (c) to examine whether first born boys are at higher risk than later born boys, oestrogen levels during pregnancy being higher among primiparous than among multiparous women (Bernstein et al, 1986; Panagiotopoulou et al, 1990; Potischman and Troisi, 1999).

During a two-year period (1996–1997) 55 new cases of breast cancer were diagnosed among male permanent residents of Greater Athens (male population around 2 million). About half of these cases were diagnosed and treated in the two major cancer hospitals of Greater Athens 'Agios Savas' and 'Metaxas' and all 23 histologically confirmed cases were included in this study.

For every male patient with breast cancer we attempted to identify four controls matching for age (± five years), two visitors in the same hospital and two injured patients in the single trauma hospital of Athens (KAT). All controls had to be free from a history of cancer at any site and be permanent residents of Greater Athens. No second visitor control was identified for eight cancer cases, whereas eight potential controls (four hospital visitors and four trauma patients) refused to be interviewed. Thus, the study included 23 male breast cancer patients and 76 controls. Each case had at least two controls. All cases and controls were interviewed by a board qualified surgeon (G.G.), who, unavoidably, was aware of the case or control status of each subject. The questionnaire covered sociodemographic and life-style characteristics, as well as regular use of medications (excluding dietary supplements). In addition, all 99 participants were asked to indicate average frequency of orgasms per month, during different periods of their life.

As visitor controls and trauma patients were similar with respect to all the studied variables, the 76 controls were combined in a single group and compared with the 23 cases. Univariate analyses showed that sibship size was positively associated with birth order, as were alcohol consumption, tobacco smoking and coffee drinking. However, mutual adjustment, as necessary, through logistic regression, did not notably change the relevant effect estimates. Given the small number of cases and the apparent lack of confounding we decided to present simple, straightforward analyses, using modelling only when there was a need to document absence of substantial confounding.

Cases and controls showed similar distributions with respect to age, family status, number of own children, weight at diagnosis and sibship size. In contrast, there was a statistically significant inverse association between birth order and breast cancer risk. Sibship size did not appreciably confound the association between birth order and breast cancer (Table 1). No evidence was found for an effect on breast cancer risk of any occupational exposure, although the numbers were small (Table 2).

*Received 6 June 2000*
*Revised 27 July 2000*
*Accepted 27 July 2000*

*Correspondence to:* Dimitrios Trichopoulos

**Table 1** Distribution of 23 breast cancer cases and 76 age-matched controls by sociodemographic variables

| Variable | Cases (*n*) | Controls (*n*) | Odds ratio (95% CI)[a] | *P* value 2-tailed[b] |
|---|---|---|---|---|
| Age (years) | | | | |
| ≤ 49 | 2 | 12 | Matched variable | 0.42 |
| 50–59 | 4 | 14 | | |
| 60–69 | 8 | 24 | | |
| ≥ 70 | 9 | 26 | | |
| Sibship size | | | | |
| ≤ 2 | 13 | 28 | Baseline | 0.15 |
| ≥ 3 | 10 | 48 | 0.45 (0.15–1.28) | |
| Birth order | | | | |
| 1 | 10 | 18 | Baseline | 0.02 |
| 2 | 7 | 17 | 0.74 (0.19–2.77) | |
| ≥ 3 | 6 | 41 | 0.26 (0.07–0.96) | |
| Marital status | | | | |
| Married/widowed | 21 | 72 | Baseline | 0.92 |
| Single/divorced | 2 | 4 | 1.71 (0.14–12.87) | |
| Number of children | | | | |
| 0 | 4 | 12 | Baseline | 0.89 |
| ≥ 1 | 19 | 64 | 0.89 (0.23–4.24) | |
| Weight (kg) | | | | |
| ≤ 64 | 2 | 12 | Baseline | 0.89 |
| 65–69 | 5 | 9 | 3.33 (0.40–40.90) | |
| 70–74 | 6 | 16 | 2.25 (0.32–26.07) | |
| ≥ 75–79 | 10 | 39 | 1.54 (0.27–16.26) | |

[a]95% Confidence Intervals (CI) calculated using exact methods. [b]From $\chi^2$ with one degree of freedom for 2*2 tables with Yates' correction; and from $\chi^2$ for trend for 2*k tables.

**Table 2** Distribution of 23 breast cancer cases and 76 age-matched controls by history of occupational exposures

| Variable | Cases (*n*) | Controls (*n*) | Odds ratio (95% CI)[a] | *P* value 2-tailed |
|---|---|---|---|---|
| Ionizing radiation | | | | |
| No | 23 | 75 | Baseline | 0.41 |
| Yes | 0 | 1 | Undefined | |
| Pesticides | | | | |
| No | 15 | 48 | Baseline | 0.95 |
| Yes | 8 | 28 | 0.91 (0.30–2.66) | |
| Oil products | | | | |
| No | 20 | 67 | Baseline | 0.83 |
| Yes | 3 | 9 | 1.12 (0.18–5.06) | |
| Elevated temperature | | | | |
| No | 17 | 54 | Baseline | 1.00 |
| Yes | 6 | 22 | 0.87 (0.25–2.71) | |
| Dust (any type) | | | | |
| No | 15 | 58 | Baseline | 0.43 |
| Yes | 8 | 18 | 1.72 (0.54–5.18) | |

[a]95% Confidence Intervals (CI) calculated using exact methods.

One case and four controls indicated that they were primarily homosexual, essentially similar proportions. Table 3 shows the number of reported orgasms per month at ages 20–29 years (all subjects) and 50–59 years (those aged 50 and over). There is an inverse association between frequency of orgasm and male breast cancer risk, stronger and highly significant at age 50–59 years.

In Table 4, the results concerning life-style variables are presented. Coffee and alcohol intake show no relation to risk. On the contrary, tobacco smoking is inversely associated with this risk. Several drugs were reported by participants as being regularly taken and for two, results are shown in Table 4. Aspirin was the regularly taken drug most frequently reported and intake of more than seven aspirin tablets per month was inversely (but not significantly) associated with risk. The statistically significant positive association with tranquillizer intake is probably a chance finding given the multiple comparisons. Three cases and two controls reported longstanding gynaecomastia, an OR of 5.55 (95% CI 0.58–69.09). The results remained essentially unchanged when several multiple logistic regression models were examined (data not shown).

There have been at least two much larger studies of male breast cancer (Thomas et al, 1992; Hsing et al, 1998) and several others with statistical power similar to that of ours (Mabuchi et al, 1985; Casagrande et al, 1988; Lenfant-Pejovic et al, 1990; D'Avanzo and La Vecchia, 1995; Stenlund and Floderus, 1997). In the present small study however, we have examined two new hypotheses and for both, plausible and statistically significant findings were generated. Frequency of orgasms, possibly because its validation as a correlate of DHT, the hormone which probably drives male sexual behaviour, is only recent (Mantzoros et al,

*© 2000 Cancer Research Campaign* 

**Table 3** Distribution of 23 breast cancer cases and 76 age-matched controls by number of orgasms per month at the age of around 25 (all subjects) and around 50 (subjects 50 years or older)

| Number of orgasms per month | Cases (*n*) | Controls (*n*) | Odds ratio (95% CI)[a] | *P* value 2-tailed[b] |
|---|---|---|---|---|
| Men 20–29 years | | | | 0.08 |
| ≤ 6 | 11 | 18 | Baseline | |
| 7–13 | 5 | 27 | 0.30 (0.09–1.00) | |
| ≥ 14 | 7 | 31 | 0.37 (0.12–1.11) | |
| Men 50+ years | | | | 0.004 |
| ≤ 6 | 13 | 14 | Baseline | |
| 7–13 | 6 | 37 | 0.18 (0.06–0.53) | |
| ≥ 14 | 2 | 13 | 0.17 (0.03–0.81) | |

[a]95% Confidence Intervals (CI) calculated using exact methods. [b]From $\chi^2$ for trend for 2*k tables.

**Table 4** Distribution of 23 breast cancer cases and 76 age-matched controls by lifestyle variables and regular use of specific medicines

| Category | Cases (*n*) | Controls (*n*) | Odds ratio (95% CI)[a] | *P* value 2-tailed[b] |
|---|---|---|---|---|
| Lifestyle | | | | |
| Smoking | | | | |
| Never | 9 | 20 | Baseline | 0.03 |
| Ex-smoker | 11 | 25 | 0.98 (0.30–3.25) | |
| Smoker | 3 | 31 | 0.22 (0.03–1.02) | |
| Coffee drinking | | | | |
| No | 6 | 14 | Baseline | 0.62 |
| Yes | 17 | 62 | 0.64 (0.19–2.36) | |
| Alcohol consumption | | | | |
| None | 4 | 10 | Baseline | 0.12 |
| Occasional drinker (< 7/week) | 12 | 26 | 1.15 (0.26–6.07) | |
| Regular drinker (≥ 7 week) | 7 | 40 | 0.44 (0.09–2.48) | |
| Regular use of medicines | | | | |
| Aspirin tablets/month | | | | |
| 0 | 15 | 44 | Baseline | 0.39 |
| 1–6 | 7 | 24 | 0.86 (0.26–2.62) | |
| ≥ 7 | 1 | 8 | 0.37 (0.01–3.18) | |
| Tranquillizer(s) | | | | |
| No | 17 | 72 | Baseline | 0.01 |
| Yes | 6 | 4 | 6.35 (1.31–33.35) | |
| Medical condition | | | | |
| Gynaecomastia | | | | |
| No | 20 | 74 | Baseline | 0.15 |
| Yes | 3 | 2 | 5.55 (0.58–69.09) | |

[a]95% Confidence Intervals (CI) calculated using exact methods. [b]From $\chi^2$ with one degree of freedom for 2*2 tables with Yates' correction; and from $\chi^2$ for trend for 2*k tables.

1995). Also, the association with birth order (Hsieh et al, 1991; Adebamowo and Adekunle, 1999) and its possible mechanism (Bernstein et al, 1986; Panagiotopoulou, 1990) have been previously evaluated only among women.

Despite the small size, the present study has some methodological strengths. Although the interviews were not blind to case-control status, they were conducted by the same medically qualified person in-hospital, and there were few refusals. Both visitor and trauma patient controls showed similar findings, as in earlier studies in Greece (Kalapothaki et al, 1993). Also, the established risk factors for male breast cancer, namely an increased risk among men who were single, childless, or with gynaecomastia were confirmed in this study, although none of these associations was statistically significant perhaps because of the small numbers.

Many previous studies have indicated that male breast cancer is more common among men who are single or have few or no children (Schottenfeld et al, 1963; Casagrande et al, 1988; Lenfant-Pejovic et al, 1990; Thomas et al, 1992; D'Avanzo and La Vecchia, 1995; Cocco et al, 1998). These findings have been assumed to reflect reduced sexual drive (Rose, 1988; Thomas et al, 1992). Our results support this assumption, in that men with breast cancer had fewer orgasms than control participants at comparable ages. The findings could not be explained by confounding and it is unlikely due to bias; a link between reduced libido and male breast cancer has not been generally suspected by the public.

The inverse association between tobacco smoking and male breast cancer risk is not implausible given the evidence that smoking in men (as well as in women) tends to produce hypo-oestrogenic effects (Michnovicz and Fishman, 1990). An inverse, but statistically non-significant, association between cigarette smoking and risk of male breast cancer can also be seen in the data of Mabuchi et al (1985) and Casagrande et al (1988).

Ever since Bernstein et al (1986) and Panagiotopoulou et al (1990) reported high levels of oestrogens in first rather than

 *© 2000 Cancer Research Campaign*

subsequent pregnancies, the hypothesis of prenatal origin of breast cancer (Trichopoulos and Lipman, 1992) has gained more credibility (Hsieh et al, 1991; Adebamowo and Adekunle, 1999). Our results appear to provide some evidence for prenatal or early life influences on male breast cancer risk, as well as a link with oestrogens.

Like others, we found no evidence of occupational risks (Mabuchi et al, 1985; Lenfant-Pejovic et al, 1990; Rosenbaum et al, 1994; Stenlund and Floderus, 1997), or that hot work places increased risk as has been suggested (Mabuchi et al, 1985). Our findings on history of regular drug use are also essentially negative.

This study provides evidence that reduced sexual drive is associated with male breast cancer, whereas anti-oestrogenic influences may reduce this risk. If DHT is indeed the principal hormone responsible for male sexual behaviour (Mantzoros et al, 1995; McClellan and Markham, 1999), and since serum levels of testosterone, as well as oestradiol, have been found to be higher among cases with male breast cancer than among controls (Rose, 1988), it seems reasonable to speculate that reduced conversion of testosterone to DHT underlies at least one stage of the pathogenesis of male breast cancer.

### REFERENCES

Adami HO, Signorello LB and Trichopoulos D (1998) Towards an understanding of breast cancer etiology. *Semin Cancer Biol* **8**: 255–262

Adebamowo CA and Adekunle OO (1999) Case-controlled study of the epidemiological risk factors for breast cancer in Nigeria. *Br J Surg* **86**: 665–668

Bernstein L, Depue RH, Ross RK, Judd HL, Pike MC and Henderson BE (1986) Higher maternal levels of free estradiol in first compared to second pregnancy: early gestational differences. *J Natl Cancer Inst* **76**: 1035–1039

Casagrande JT, Hanisch R, Pike MC, Ross RK, Brown JB and Henderson BE (1988) A case-control study of male breast cancer. *Cancer Res* **48**: 1326–1330

Cocco P, Figgs L, Dosemeci M, Hayes R, Linet MS and Hsing AW (1998) Case-control study of occupational exposures and male breast cancer. *Occup Environ Med* **55**: 599–604

Collaborative Group on Hormonal Factors in Breast Cancer (1996) Breast cancer and hormonal contraceptives: collaborative reanalysis of individual data on 53 297 women with breast cancer and 100 239 women without breast cancer from 54 epidemiological studies. *Lancet* **347**: 1713–1727

Collaborative Group on Hormonal Factors in Breast Cancer (1997) Breast cancer and hormone replacement therapy: collaborative reanalysis of data from 51 epidemiological studies of 52,705 women with breast cancer and 108,411 women without breast cancer. *Lancet* **350**: 1047–1059

D'Avanzo B and La Vecchia C (1995) Risk factors for male breast cancer. *Br J Cancer* **71**: 1359–1362

Hankinson SE, Willett WC, Manson JE, Colditz GA, Hunter DJ, Spiegelman D, Barbieri RL and Speizer FE (1998) Plasma sex steroid hormone levels and risk of breast cancer in postmenopausal women. *J Natl Cancer Inst* **90**: 1292–1299

Hsieh CC, Tzonou A and Trichopoulos D (1991) Birth order and breast cancer risk. *Cancer Causes Control* **2**: 95–98

Hsing AW, McLaughlin JK, Cocco P, Co Chien HT and Fraumeni JF Jr. Risk factors for male breast cancer (United States) (1998) *Cancer Causes Control* **9**: 269–275

Kalapothaki V, Tzonou A, Hsieh CC, Toupadaki N, Karakatsani A and Trichopoulos D (1993) Tobacco, ethanol, coffee, pancreatitis, diabetes mellitus, and cholelithiasis as risk factors for pancreatic carcinoma. *Cancer Causes Control* **4**: 375–382

Lenfant-Pejovic MH, Mlika-Cabanne N, Bouchardy C and Auquier A (1990) Risk factors for male breast cancer: a Franco-Swiss case-control study. *Int J Cancer* **45**: 661–665

Mabuchi K, Bross DS and Kessler II (1985) Risk factors for male breast cancer. *J Natl Cancer Inst* **74**: 371–375

Mantzoros CS, Georgiadis EI and Trichopoulos D (1995) Contribution of dihydrotestosterone to male sexual behaviour. *BMJ* **310**: 1289–1291

McClellan KJ and Markham A (1999) Finasteride: a review of its use in male pattern hair loss. *Drugs* **57**: 111–126

Michnovicz JJ and Fishman J (1990) Increased oxidative metabolism of oestrogens in male and female smokers. In: Wald N, Baron J (eds) *Smoking and Hormone-Related Disorders*. Oxford University Press: Oxford, pp 197–208

Panagiotopoulou K, Katsouyanni K, Petridou E, Garas Y, Tzonou A and Trichopoulos D (1990) Maternal age, parity, and pregnancy estrogens. *Cancer Causes Control* **1**: 119–124

Potischman N and Troisi R (1999) In-utero and early life exposures in relation to risk of breast cancer *Cancer Causes Control* **10**: 561–573

Rose DP (1988) Endocrine epidemiology of male breast cancer (Review). *Anticancer Res* **8**: 845–850

Rosenbaum PF, Vena JE, Zielezny MA and Michalek AM (1994) Occupational exposures associated with male breast cancer. *Am J Epidemiol* **139**: 30–36

Schottenfeld D, Lilienfeld AM and Diamond H (1963) Some observations on the epidemiology of breast cancer among males. *Amer J Publ Hlth* **53**: 890–897

Sorensen HT, Friis S, Olsen JH, Thulstrup AM, Mellemkjaer L, Linet M, Trichopoulos D, Vilstrup H and Olsen J (1998) Risk of breast cancer in men with liver cirrhosis. *Am J Gastroenterol* **93**: 231–233

Stenlund C and Floderus B (1997) Occupational exposure to magnetic fields in relation to male breast cancer and testicular cancer: a Swedish case-control study. *Cancer Causes Control* **8**: 184–191

Thomas DB, Jimenez LM, McTiernan A, Rosenblatt K, Stalsberg H, Stemhagen A, Thompson WD, Curnen MG, Satariano W, Austin DF et al (1992) Breast cancer in men: risk factors with hormonal implications. *Am J Epidemiol* **135**: 734–748

Toniolo PG, Levitz M, Zeleniuch-Jacquotte A, Banerjee S, Koenig KL, Shore RE, Strax P and Pasternack BS (1995) A prospective study of endogenous estrogens and breast cancer in postmenopausal women. *J Natl Cancer Inst* **87**: 190–197

Trichopoulos D and Lipman RD (1992) Mammary gland mass and breast cancer risk. *Epidemiology* **3**: 523–526

*© 2000 Cancer Research Campaign*

